# Gene therapy for cardiovascular disease mediated by ultrasound and microbubbles

**DOI:** 10.1186/1476-7120-11-11

**Published:** 2013-04-17

**Authors:** Zhi-Yi Chen, Yan Lin, Feng Yang, Lan Jiang, Shu ping Ge

**Affiliations:** 1 Department of Ultrasound Medicine, Key Laboratory for Major Obstetric Diseases of Guangdong Province, The Third Affiliated Hospital of Guangzhou Medical University, Guangzhou, 510150, China; 2 Section of Cardiology, St. Christopher’s Hospital for Children, Drexel University College of Medicine, 3601 A Street, Philadelphia, PA, USA

**Keywords:** Ultrasound, Microbubble, Gene therapy, Cardiovascular

## Abstract

Gene therapy provides an efficient approach for treatment of cardiovascular disease. To realize the therapeutic effect, both efficient delivery to the target cells and sustained expression of transgenes are required. Ultrasound targeted microbubble destruction (UTMD) technique has become a potential strategy for target-specific gene and drug delivery. When gene-loaded microbubble is injected, the ultrasound-mediated microbubble destruction may spew the transported gene to the targeted cells or organ. Meanwhile, high amplitude oscillations of microbubbles increase the permeability of capillary and cell membrane, facilitating uptake of the released gene into tissue and cell. Therefore, efficiency of gene therapy can be significantly improved. To date, UTMD has been successfully investigated in many diseases, and it has achieved outstanding progress in the last two decades. Herein, we discuss the current status of gene therapy of cardiovascular diseases, and reviewed the progress of the delivery of genes to cardiovascular system by UTMD.

## 

With the rapid development of economy, urbanization and changing lifestyles, the number of people with cardiovascular diseases is increasing globally [1]. Even if recent progress have been made in diagnosis and remedy, cardiovascular disease remains the leading cause of mortality in many countries [2]. Therefore, there is a strong impetus for more effective treatment and prevention. As increasing insight into the molecular mechanisms of cardiovascular diseases, gene therapy has been proposed as a promising therapeutic tool for the treatment of cardiovascular diseases [3,4].

To realize the therapeutic effect, both efficient delivery to the target cells and sustained expression of transgenes are required. In recent years, a large number of proof-of-principle studies have confirmed that ultrasound targeted microbubble destruction (UTMD) could enhance transfection efficiency of naked plasmid DNA by several orders of magnitude [5-7]. Therefore, it is considered as a promising strategy for gene therapy. Herein, we discuss the current status of gene therapy of cardiovascular diseases and review the studies of gene therapy of cardiovascular diseases mediated by UTMD.

## Barriers of gene therapy for cardiovascular diseases

To realize efficient delivery of therapeutic genes to the cardiovascular system, a series of barriers related to almost all aspects of cellular biology have to be overcome. Firstly, the gene vectors need to pass through the endothelial barriers in capillary walls when systemically injected. Meanwhile, the plasmid faces a threat of being degraded rapidly by the immune system or DNAse in serum before transfection. On the other hand, viral gene vectors need to avoid the immunoreaction in circulation and transduction of non-target organs, mainly liver and spleen. Secondly, as gene vectors and plasma membrane are negatively charged, the gene vectors have to diffuse through myocardial membrane then bind to cell surface but to be repelled from it. Thirdly, the plasmid needs to avoid being entrapped into lysosome or the endosome, where it will be degraded. Fourthly, the gene vector has to penetrate the nuclear membrane to achieve the goal of gene therapy. Nevertheless, appropriate technologies can be used to make the gene vector itself complete target to the interested area, such as injection catheter, surgical operation or UTMD [8,9]. Also directly injection of the vector into myocardium will lead to high local concentration of the vector. Optimized surface of the vector can realize directional transduction of the vector into the cell and karyon.

## Current status of gene therapy of cardiovascular disease

Nabel *et al.*[10] were the first to demonstrate gene therapy in cardiovascular system in 1989. Since then, gene therapeutic trials for cardiovascular diseases have been performed all over the world. However, advances in the area of gene therapy for cardiovascular disease are not well satisfied because of the lack of gene delivery systems to transfer therapeutic gene to specific target to provide an adequate dose of a therapeutic gene [2]. So far, the gene delivery systems are mainly divided into two kinds, namely, viral systems and non-viral systems [11].

Viral systems derived from adeno-associated virus (AAV) [12], retrovirus [13], lentivirus [14] and adenovirus [15] are one of the successful gene delivery systems, which are used in the majority of current gene therapy researches and clinical trials due to their benefits of highly efficient delivery into cells with sustained expression. Recently, Prunier *et al.*[16] showed that delivering adenovirus expressing sarcoplasmic reticulum Ca^2+^ ATPase (SERCA2a) into coronary arteries could prevent ventricular arrhythmias in a ischemia - reperfusion model. Suckau *et al.*[17] also employed adenoviral and AAV vectors to obtain high RNAi activity. They showed that an adenoviral short hairpin RNA vector could silence phospholamban in cardiomyocytes and improved hemodynamics in heart-failure. Meanwhile, they designed a dimeric cardiotropic AAV vector to intravenously deliver RNA molecule to the heart for simplified long-term therapy. However, viral gene therapy has been subjected to criticism due to their potential for uncontrollable and insertional mutagenesis [18]. Moreover, viral vectors will evoke undesired immune response by the virtue of systemic administration, which limits repetitive regiments [19,20]. Also the transfection efficiency of viral vectors occur with a relatively low efficiency and organ specificity, which restricts its therapeutic efficacy [21,22].

Nonviral systems is consist of chemical methods (such as cationic liposome, nanoparticle and polymers) and physical methods (include gene gun, electroporation, particle bombardment, ultrasound utilization, and magnetofection) [23,24]. The advantages of nonviral system include availability, cost-effectiveness, and less induction of immune system and no limitation in size of transgenic DNA compared with viral system, which have made them an attractive candidate for gene delivery. Among them, the simplest and most widely nonviral gene vector is naked DNA. However, its transfection efficiency is limited due to the rapid degradation by DNAse and the clearance by the mononuclear phagocyte system in the systemic circulation. Increasing target specificity to diseased tissue can reduce off-target bioeffects and enhance the gene transfection efficiency. Recently, Ko et al. [25] conjugated cell-penetrating transactivating transcriptional activator (TAT) peptide (TATp) and/or monoclonal anti-myosin monoclonal antibody 2G4 (mAb 2G4) which target to cardiac myosin to liposomes for targeted gene delivery to ischemic myocardium. The result showed that in vitro transfection was enhanced by the presence of TATp and was further enhanced by the additional modification with mAb 2G4 antibody. And the transfection efficiency of in vivo experiments was significantly enhanced in the ischemic zone. The main disadvantage of nonviral systems is their low transfection because of the inability of vectors to overcome biological barriers to enter cells [26]. In other hand, physical methods recently have been developed as a feasible nonviral method. However, most of them are mainly based upon invasion procedures, their inherent risks that may outweigh their benefits, which makes them inappropriate for cardiac gene transfer. Durieux et al. [27] conducted a study to investigates the factors regarding gene electrotransfer associated muscle damage, which confirmed that gene electrotransfer associated muscle damage was related to the intracellular presence and expression of plasmid DNA. Besides, the gene expression is confined to the injection site. Therefore, it is necessary to develop an effective and specific gene delivery system suitable for human body [28].

UTMD has been widely proved as a new strategy of improving the delivery of drugs or genes [29,30]. Due to its advantages of high safety profile, repetitive applicability, cost-effectiveness and the capability to enhance the permeability of plasma membrane to macromolecules by its bioeffects, it is considered as a feasible tool for gene delivery [31-35]. Chen *et al.*[36] have proved that UTMD significantly increased the transfection rate of short hairpin RNA (shRNA) vectors in vitro and in vivo, which was equal to that of some cancer cell lines delivered by polyethylenimine (PEI). Qiu *et al.*[37] have proved that enhanced green fluorescent protein (EGFP) plasmids could be delivered effectively into rabbit Achilles tendons by UTMD, causing no obvious injury.

## Mechanisms of UTMD in gene therapy

**Figure 1:**
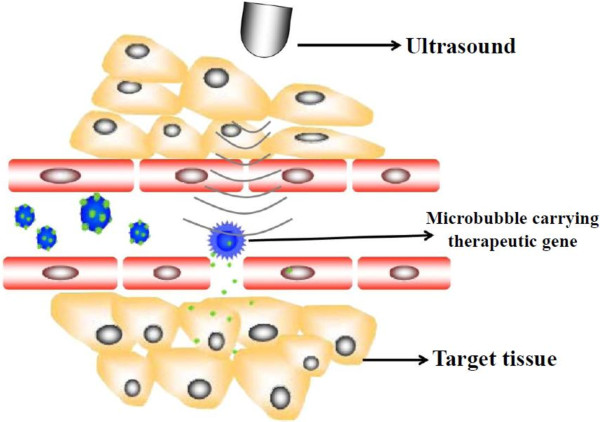
**Schematic diagram of gene therapy mediated by UTMD.** Microbubbles carrying therapeutic gene are destroyed at the site of the target tissue, resulting in sonoporation and delivery of the drug directly to the target cell. The process of sonoporation induced by US application leads to transiently holes in cell membrane and capillary, which facilitates the uptake of therapeutic gene.

UTMD is an immense potential target-specific gene delivery tool. Its ability to elevate the gene transfection efficiency in various studies in vitro and in vivo has been confirmed [38-40], thus being consider as a promising gene carrier approach for gene therapy. Microbubbles(MBs) of UTMD, which may consist of lipids, albumin, saccharide, biocompatible polymers and other materials [41-43] are traditionally used as ultrasound contrast agents due to their physical property of reflecting ultrasound. Microbubble as cavitation nucleus could expand and contract under the effect of ultrasound, and even be disrupted when the acoustic pressure reaches a much higher level, which could cause a series of biological effects. The mechanism of transferring gene into cells effectively through UTMD is based on the specific response of the microbubbles upon exposure to ultrasound, namely sonoporation. Microbubbles may oscillate when exposed to ultrasound, and then these oscillating microbubbles may rupture. So, the gene therapy vector incorporated with microbubbles can be released with high local concentrations at the site of interest. Meanwhile, the destruction of MBs may transiently induce transient holes in membranes in consequence to local shear forces exerted on membranes by fluid flow (‘micro streaming’) around oscillating bubbles, local shock waves (which produce large pressure gradients across a cell), or cavitation microjets, therefore, facilitating drug or gene into cells [44,45], and augmenting the transfection efficiency (Figure 1). The advantages of UTMD techniques are as follows: (1) low toxicity, (2) low immunogenicity of the vectors, (3) low invasiveness (e.g., the vector and MB can be intravenously injected), (4) great potential for repetitive application, and (5) organs can be targeted with high specificity. Since UTMD can not only improve the efficiency, but also avoid the immunogenicity, it has been regarded as a new choice for gene therapy.

## Gene therapy mediated by UTMD in cardiovascular disease

Due to its advantages of high safety, cost effectiveness, repetitive applicability, and the possibility to increase the permeability of microvessel and plasma membrane to macromolecules by its bioeffects, ultrasound and microbubble has been considered as a powerful tool in gene therapy. In recent years, many studies in vitro and in vivo have confirmed that ultrasound and microbubble could significantly elevate the gene transfection efficiency. It is emerging as a potential strategy for treatment of cardiovascular diseases [11].

### Delivery genes to cardiovascular system by microbubble and ultrasound

Ultrasound and microbubble have been widely investigated in myocardial infarctions, atherosclerosis, and in hind limb ischemia models in rodents for therapeutic angiogenesis. Early in the last decade, commercial and custom microbubbles were tested for pDNA encoding luciferase delivery in the left ventricle [46].

**Figure 2:**
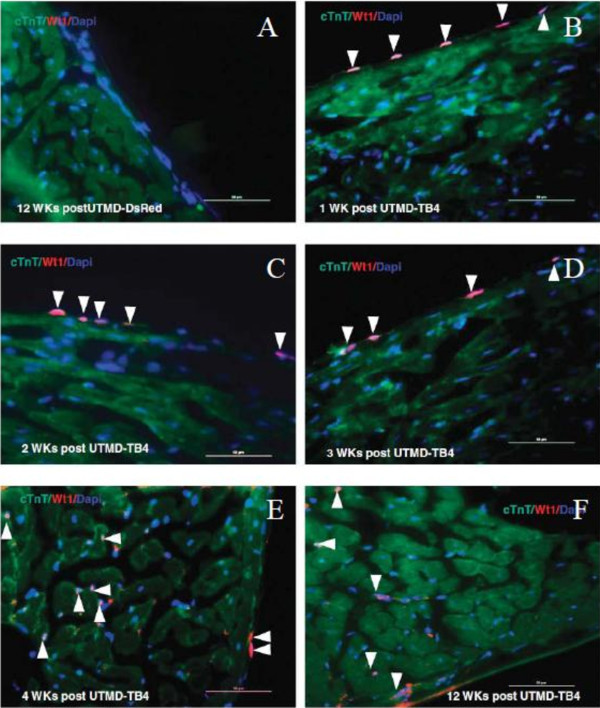
**Exogenous TB4 reprogrammed epicardium layer cells into WT1-positive adult cardiac progenitor cells and then formed new cardiac muscle cells. A**: Negative control for 12 weeks post-UTMD-DsRed;**B**-**F**: The panels are high power images (scale bar = 50 mm) from TB4-treated rats killed at 1, 2, 3, 4 and 12 weeks after UTMD. WT1 signal from nucleus of epicardium layer cells at 1–3 weeks after UTMD-TB4 treatment and then migrated into myocardium layer to form new cardiac muscle cells (WT1 signal from nucleus of cTnT-positive cells, arrows) at 4 or 12 weeks after UTMD-TB4 treatment [47].

It has proved that TFPI-2 played an important role in suppressing thrombosis and arterial re-stenosis, which has been considered as a potential gene for gene therapy of atherosclerosis. Studies have confirmed that TFPI-2 gene can be delivered to the target specifically by the virtue of UTMD. For example, Wang *et al.*[39] showed gene transfection with SonoVue and TFPI-2 gene could suppress thrombosis and arterial re-stenosis, providing a potential gene therapy approach for atherosclerosis. Compared with adenovirus, the in vivo transfection efficiency of SonoVue was higher than that of adenovirus and SonoVue was less damaging when transfecting genes into the arterial wall. Studies have confirmed that thymosin beta 4 (TB4)-protein delivery could stimulate differentiation of resident adult WT1-positive (WT1 is a biomarker for development of embryonic heart and is not normally expressed in adult rat heart) cardiac progenitor cells, but its application is limited due to low efficiency. Chen *et al.*[47] used UTMD to enhance the delivery of the human TB4 gene under a piggybac transposon plasmid to normal rat heart. The result showed that WT1 started to express from the nucleus of epicardium layer cells one week after UTMD-TB4 treatment (Figure 2), and the level of WT1 mRNA of which treated with UTMD-TB4 group was 43.5-fold higher than in normal control or UTMD-DsRed groups in the 4th week, and c-kit mRNA level in UTMD-TB4 group was 52-fold higher after UTMD-TB4 treatment.

**Figure 3:**
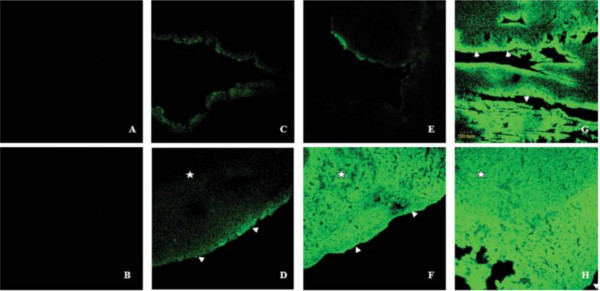
**The combination of UTMD and PEI *in vivo*. A**, Phosphate-buffered saline group; **B**, phosphate-buffered saline + US; **C**, naked plasmid; **D**, plasmid + US; **E**, plasmid + liposome microbubbles (LM); **F**, plasmid + LM + US; **G**, plasmid + LM + PEI; **H**, plasmid + LM + PEI + US. There were no fluorescent signals in the negative control groups (**A**, **B**). When the mouse hearts exposed, the bases of the heart tissue samples had more transfected cells than the rest of the samples (**D**-**H**). Only a few fluorescent signals could be detected in the absence of US (**E**). A transmural fluorescent signal could be observed in the anterior wall after the microbubble was destroyed (**F**). EGFP was principally expressed in the subendocardial layer in the absence of US(**C**, **E**, and **G** and arrows in **G**). Combining with PEI and UTMD, the distribution of EGFP was not significant (**H**) [5].

Chen *et al.*[5] have proved that the combination of UTMD with PEI could effectively enhance transfection efficiency of two different naked DNA without causing any apparently side effect (Figure 3). Besides, they demonstrated that naked plasmids (luciferase reporter, red fluorescent protein reporter, EGFP) could be effectively delivered to myocardium, combining with liposome microbubble (MB), PEI and ultrasound (US). However, though UTMD-mediated naked DNA in gene therapy is effective, this technology has some limitations. The parameters for this technique, including the US exposure parameters, US frequency, mode of US, mechanical index, and amount of the plasmid DNA, should be optimized in the future.

### Delivery viral vectors to cardiovascular system by ultrasound and microbubble

Due to its high transfection efficiency and sustained expression, viral vector is the priority choice of transferring genes to the target cell. However, it is difficult to restrict the specificity of delivery in viral vectors which are usually delivered systemically, while avoid the immunoreaction. Additionally, the endothelial barrier limits systemic delivery of viral vector such as AAV, which leads to unsatisfactory transfection efficiency. UTMD techniques have many advantages [48]. Studies have proved that it had synergism to combine with viral vectors, which offered many benefits [49,50]. Firstly, the microbubbles offer the strength of site-specific release through ultrasound irradiation, thus improving viral vector specificity. And the production of microjet by UTMD can enhance the penetrability of plasma membrane and capillary, thus overcoming the endothelial barrier. Moreover, the microbubbles can simultaneously impose restriction on the immune response to the viruses thus allowing intravascular administration and repetitive injections [51].

Recently, a study by Naka *et al.*[52] demonstrated that gene expression mediated by retrovirus was significantly increased in all four cell types tested in this study without any adverse bioeffecs when they were exposed for 5 s with the ultrasound of 1.0 W/cm^2^. The transduction efficiencies of ultrasound was enhanced 6.6-fold, 4.8-fold, 2.3-fold, and 3.2-fold in 293T cells, BAECs, RASMCs, and L6 cells, respectively. Furthermore, in the presence of ultrasound and the retrovirus, β-Gal activities of these cells were also increased. Chen *et al.*[46] optimized echocardiographic parameters for successfully delivered adenoviral or plasmid DNA to the heart. The results demonstrated that enhancement of transgene expression are detected in the heart tissue when treated with UTMD associating with adenoviral or plasmid DNA. And the ultrasound parameters were optimized with a low-transmission frequency (1.3 MHz), maximal mechanical index, and ECG triggering to allow completely fitting the myocardial capillary bed for microbubbles dispose. In addition, Lee *et al.*[53] showed that ultrasonic standing wave fields could offer a potential approach to increase transduction efficiency of retrovirus-based vector in large-scale settings.

**Figure 4:**
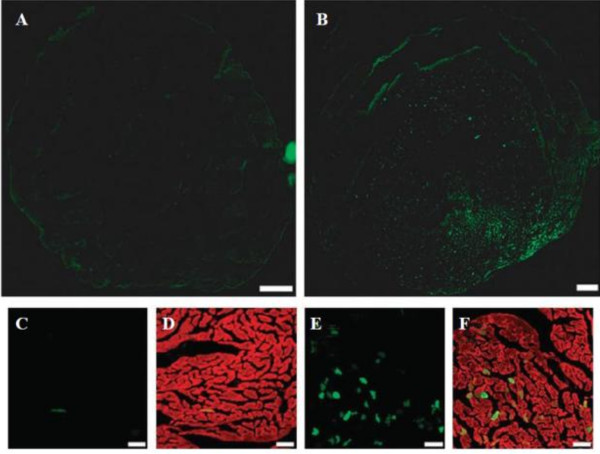
**Localization of gene expression after treatment with UTMD.** Adult rats were injected with microbubbles loaded with 10^11^ genomic particles of AAV-9-cytomegalovirus (CMV)-MLC0.26-EGFP vectors. (**A**) No fluorescent signals were detectable in the absence of ultrasound; (**B**) The anterior wall showed a strong fluorescent signal after UTMD; (**C**) When is amplified, only few fluorescent signals could be observed in the absence of microbubbles; (**D**) The merged picture of green fluorescent protein (GFP)-fluorescence and phalloidin co-staining; (**E**) In the presence of UTMD, multiple GFP-postive cells can be observed; (**F**) co-staining with phalloidin. Bar a, b: 1 mm and c ~ f: 50 μm [49].

Since, systemic administration of these viruses had been a challenge as the drawback of adenoviral vectors has innate host antiviral immune responses. Howard *et al.*[51] have tested the ability of microbubble to load and protect an adenoviral vector. The result demonstrated that systemic delivery of the vector incorporated into microbubbles led to specific targeting of the GFP transgene. Microbubbles allow intravenous injection as it can reduce the degradation rate of viruses by immune system. This opens up a new therapeutic frontier for patients needs for a less invasive and highly specific gene delivery system. Müller *et al.*[49] also conducted a study to research the specificity and transfection rate of gene delivery. In their study, adult rats were injected of microbubbles loaded with AAV-6 or AAV-9 expressing luciferase or EGFP. The result demonstrated that the reporter gene transfection efficiency significantly increased with ultrasound exposure (Figure 4).

Also, Taylor *et al.*[54] carried out a study to assess the feasibility of gene delivery system that combined UTMD and retrovirus. In their study, they added an envelope-deficient retroviral vector which was inherently incapable of target cell entry to target cells with ultrasound exposure of pulsed 1 MHz for 5s. By using virus-loaded microbubbles, ultrasound facilitated the delivery of viral vectors in a restricted area of cells exposed to ≥ 0.4 MPa peak-negative acoustic pressure. It showed that the technology was an ideal mean suited for targeted delivery.

The mechanism how ultrasound enhances the transfection rate of transgene may be that ultrasound imparts a microjet to the cell and facilitates local delivery of the DNA into the cell. It also presumedly that ultrasound affected cell regulatory or transcription factors. However, the precise mechanism accounting for why expose ultrasound to cells together with and also to viral vector enhance the gene expression level is still blurred. Specific studies should be conducted to explain these different mechanisms and decide how UTMD influences AAV-mediated gene expression. Therefore, Geers *et al.*[55] have undertaken a study to interpret the mechanisms behind how the combination of UTMD and AAV mediated gene therapy work. They made use of “non-active AAV”, which are AAV vectors chemically modified at their surface with a poly (ethylene glycol) (PEG) brush, and found the “non-active AAV” could be delivered in the cytosol of cells directly through sonoporation. Thus UTMD can specifically and effectively increase rAAV gene delivery system, and it may afford potential for highly effective gene delivery means for gene therapy of cardiovascular diseases.

### Delivery gene to cardiovascular system by ultrasound and novel microbubble

**Figure 5:**
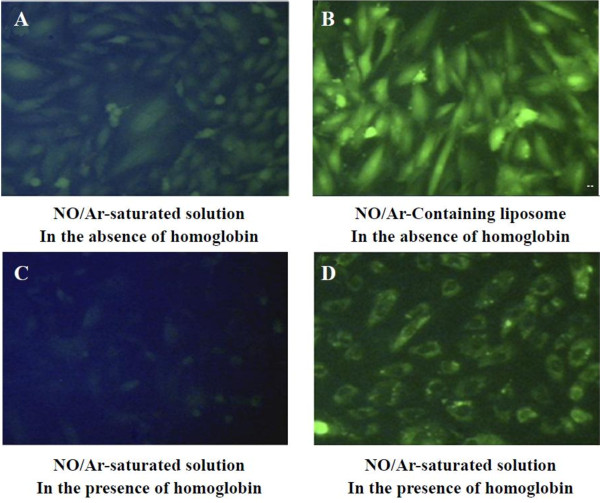
**Nitric oxide (NO) was delivered to the cultured vascular smooth muscle cells by ELIP with or without NO-scavenging agent, haemoglobin.** Vascular smooth muscle cells were transfected with a fluorescent probe, diaminofluorescein-2 diacetate (DFA-2DA), which reacts with NO to produce a fluorescent signal. In the absence of haemoglobin, **A**: the cultured cells were treated with free NO and in the absence of haemoglobin; **B**: the cultured cells were treated with NO encapsulated in ELIP. In the presence of haemoglobin, **C**: the cultured cells were treated with free NO; **D**: the cultured cells were treated with NO encapsulated in ELIP. NO-loaded ELIP were able to efficiently deliver NO into cultured cells even in the presence of a potent NO-scavenging agent such as haemoglobin [57].

Compared with the other groups, the application of viral vector significantly increased transgene expression, but the toxicity and immunogenicity of the viral vectors still plagued people. The non-viral vectors can enhance the gene transfection rate. Noninvasive UTMD enable the successful transfection of vascular endothelial grow factor (VEGF), stem cell factor or other genes into the infracted heart, thus increasing density of blood vessel, myocardial perfusion and ventricular function. UTMD-mediated plasmid gene delivery takes advantage of myocardial contrast echocardiography and has numerous merits including low toxicity, lack of immunogenicity, and the potential for repetitive and targeted application [56,57]. However, there were some injured endothelial cells in part of the vessel wall with the ultrasound exposure. Also, Huang *et al.*[57] developed new liposomes that could be used to protect and transfer a bioactive gas to target in conjugate with noninvasive UTMD. The results showed that it hold potential for gas delivery and could be used to control therapeutic gas release (Figure 5).

**Figure 6:**
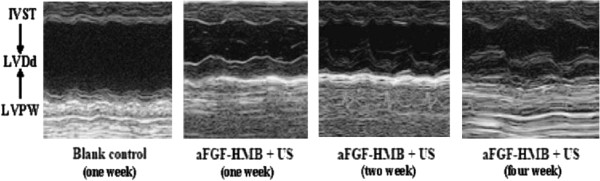
**M-mode echocardiography of Group (aFGF-HMB + US) [**[59].

Despite the existence of many in vitro experimental studies on gene therapy with combination of non-viral vectors and UTMD, research on in vivo therapeutic application is on the beginning. Saliba *et al.*[58] developed an interesting safe method for local gene transfer by injection of plasmid or siRNA mixed with a standard commercial liposome, with the presentence of ultrasound application (4.9MHz, 1Hz, 1W/cm^2^, ultra-harmonic mode, 5min, another 5min), resulted in much higher transfection efficiency. Zhao *et al.*[59] investigated that the cardio protective effect of the acidic fibroblast growth factor (aFGF) combing with heparin modified microbubbles (aFGF-HMB) under UTMD technique. Echocardiography of the heart parenchyma was enhanced after aFGF-HMB injection. From ultrasonography, aFGF-HMB suspension had good capability in heart ultrasonic contrast imaging. As shown in M-mode echocardiography, the group (aFGF-HMB + US) could remarkably stimulate myocardial vessel neogenesis, resulting in a significant improvement of regional as well as global myocardial contractile functions (Figure 6).

**Figure 7:**
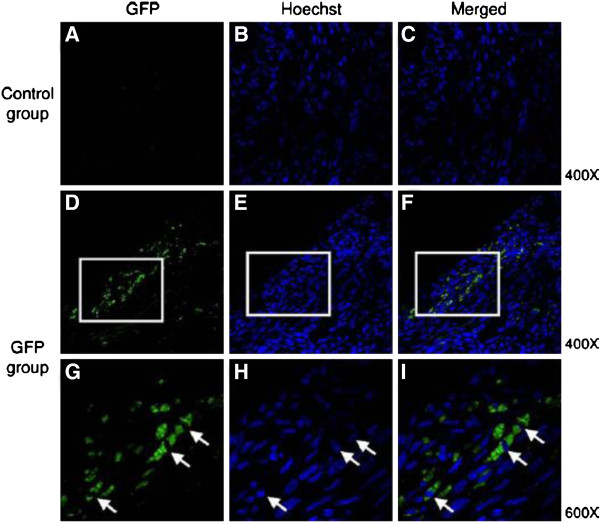
**Confocal microphotograph of myocardial tissue of mice after treatment to show the efficiency of gene transfection in vivo by UTMD.** 5 days after the treatment of UTMD in the myocardial tissue of mice, the samples were immunostained with an antibody against green fluorescent protein (GFP, green), nuclear staining (Hoechst, blue). **A** ~ **C**: control group, the mice was injected with empty plasmids; **D** ~ **I**: GFP group, the mice was injected with plasmids expressing GFP; **G** ~ **I**: the merged images indicated co-localization of GFP and Hoechst. Magnification _400 in A to F. Framed areas in **D** to **F** are shown enlarged at _600 in **G** to **I**[41].

**Figure 8:**
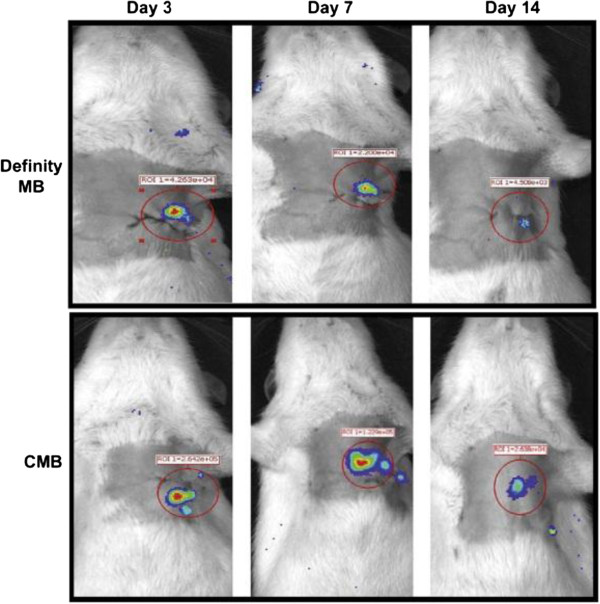
**Images of rats receiving luciferase plasmid delivered by UTMD.** Bioluminescence was significantly higher at 3, 7, and 14 days after gene delivery with the CMB compared with the Definity MB [60].

Recently, Fujii *et al.*[41] used UTMD to deliver angiogenic genes. The result showed that GFP expression was identified in the hearts of mice that were injected with microbubble/GFP expressing plasmid complex five days after UTMD and confirmed that successful in vivo transfection was achieved by using the microbubble technique (Figure 7). Sun *et al.*[60] prepared a cationic microbubble with DNA-binding to improve targeted gene transfection of the ischemic heart for cardiac regeneration. Compared its DNA-carrying capacity and reporter gene transfection efficiency with the commercially available Definity microbubble, the cationic microbubbles loaded 70% more plasmid DNA than the Definity microbubbles and UTMD was able to deliver the therapeutic gene to the ischemic rat myocardium and evaluated the effects on apoptosis, angiogenesis, and cardiac function, and provided an efficient platform for gene therapy of the ischemic heart and preserve cardiac function (Figure 8).

**Figure 9:**
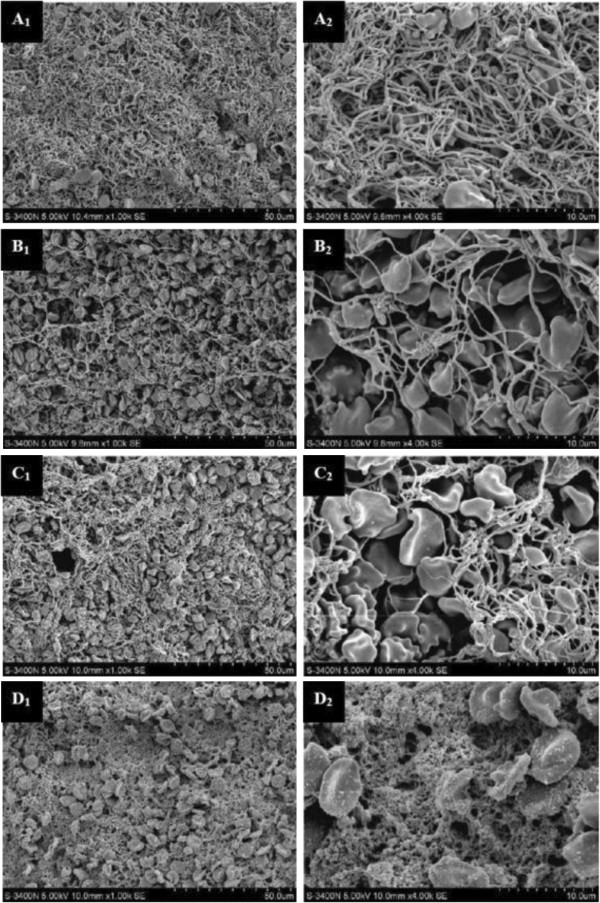
**After treatment, the thrombi were observed under the electron microscope. A1**, **A2**: control group; **B1**, **B2**: uPA group; **C1**, **C2**, **C3**, **C4**: uPA + US group; **A1**, **B1**, **C1**, **C3** were amplified by 1000 times; **A2**, **B2**, **C2**, **C4** were amplified by 4000 times [64].

Atherosclerosis is an inflammatory disease of the vasculature and risks, which can lead to heart attacks and stroke, so there is a great need for both drug delivery and detection of disease state. Phillips *et al.*[61] developed the novel microbubbles targeted to vascuolar cell adhesion molecule 1 (VCAM-1), which can be used for simultaneous ultrasound molecular imaging and gene delivery. Compared with nontargeted microbubbles, VCAM-1-targeted microbubbles exhibited a 100-fold increase in adhesion to inflamed SMCs. Their studies may aid in the detection and treatment of in-stent restenosis or be used to detect early atherosclerosis, and subsequently would realize gene or drug therapy to inflamed vasculature.

A body of laboratory work has demonstrated that UTMD was a promising tool for the local delivery of genes and drugs [62]. A novel and potential site-specific gene transfer, combination of ultrasound (1 MHz, 1.5 w/cm^2^, 10 minutes) and nanopackaged t-PA (Tissue plasminogen activator, t-PA) gene plasmid, was also employed to cure human thrombosis-related diseases. The results showed that the heart ultrasound visualization increased obviously compared to preinjection, and the baseline image following the ultrasound treatment decreased after the intravenous injection of microbubbles loaded with nanopackaged t-PA gene plasmid [63]. Additionally, Ren *et al.*[64] reported the preparation of three groups of self-made microbubble-loading uPA (1 uPA-MBs, 5 uPA-MBs and 10 uPA-MBs) via freeze-drying methods to achieve a more efficacious and safer thrombolytic effect. It can be used for thrombolysis when combined with low-frequency US in vitro. The loaded uPA exhibited bioactivity by agarose fibrin plate when exposed to US and in vitro thrombolysis also showed higher effects with US exposure related to the group who received uPA-MBs alone, the control group or the US group. In conclusion, the physiochemical properties of these self-made uPA-MBs allow for intravenous injection but 1 uPA-MB and 5 uPA-MBs are better than 10 uPA-MBs. The combination of uPA-MBs and US can minish the in vitro dosage of uPA for thrombolysis (Figure 9). Mannell *et al.*[65] loaded the magnetic (MNP) perfluorocarbon-filled lipid microbubbles with lentiviral particles and associated magnetic targeting of these complexes with UTMD. The combination eventually led to a transduction efficiency increase by 30-fold over the application of naked virus alone.

**Figure 10:**
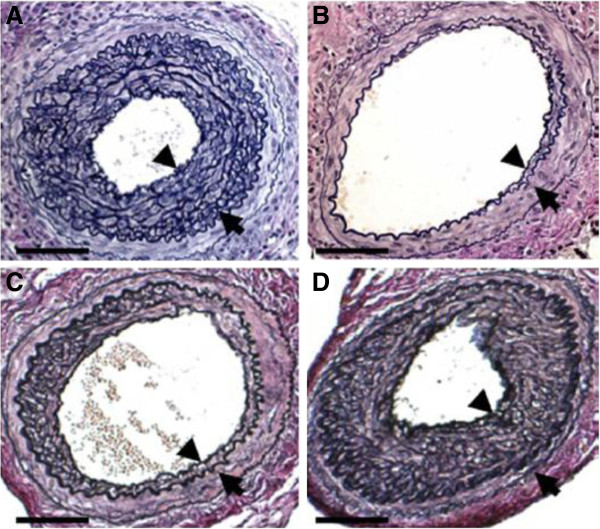
**Arteries harvested on day 28 stained with Elastica van Gieson (EvG). A**: Significantly thickened intima in the arteries is seen with ICAM-1 siRNA without ultrasound irradiation; **B**: B shows significant suppression of neointimal formation in the artery with ICAM-1 siRNA with microbubble administration and ultrasound irradiation. **C**: VACM-1 siRNA had an effect statistically comparable to that of ICAM-1 siRNA in the prevention of neointimal formation. **D**: The scrambled siRNA did not suppress neointimal formation [33].

### Application of ultrasound and microbubble in RNA interference based gene therapy for cardiovascular desease

RNA interference (RNAi) is a technique that could inhibit target gene expression based on sequence-specific gene silencing using small interfering RNA (siRNA) [66]. The technique has attracted much attention for clinical use in various diseases, and has potential to treat cardiovascular diseases. However, transfection of the endothelium and myocardial cell with siRNAs in vivo still poses a distinct hurdle [67]. There still need a noninvasive and effective method to transfer siRNA into target cells. Kinoshita *et al.*[68] demonstrated that delivery siRNA intracellularly via microbubble-enhanced focused ultrasound was viable, and represented a powerful tool for using siRNA in vivo and possibly in the clinical setting.

Among the more common approaches employed for siRNA delivery are the use of ultrasound-microbubble which can effectively delivery siRNAs into target areas in vivo. Suzuki *et al.*[33] prepared three kind of siRNA/microbubble complexes (a fluorescein-labeled siRNA, green fluorescent protein (GFP) siRNA, and intercellular adhesion molecule (ICAM)-1 siRNA), and confirmed that ICAM-1 siRNA/microbubble has the potential to suppress arterial neointimal formation using ultrasound-microbubble method (Figure 10). With ultrasound irradiation, siRNA/microbubble is potent for clinical treatment of cardiovascular diseases and other inflammatory disease.

**Figure 11:**
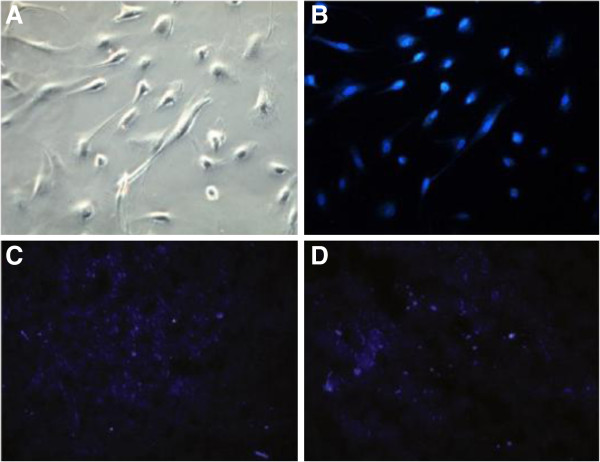
**DAPI positive cells were detected by fluorescent microscope.** Compare with MSC infusion group, the US + Microbubble + MSC group has much more DAPI-positive cells localized in the ischemic myocardium (**A**-**B**). MSCs were labeled with DAPI, and all the cells were dyed in bright blue, and observed under light microscope (×200) and fluorescent microscope. (**C**) MSCs of US + Microbubble + MSC group (×100), (**D**) MSC infusion group (×100) [56].

### Stem Cell Transplant for Treatment of Cardiovascular Diseases Mediate by Ultrasound and Microbubble

Cell-based therapy presents an attractive approach to restoration of a functional endothelium and myocardium. After acute myocardial infarction, migration of bone marrow-derived mesenchymal stem cells (MSCs) to vital myocardium is a promising therapeutic method. Ultrasound irradiation inducing stimulation of microbubbles allows locoregional pre-treatment of target tissue, and combination of ultrasound irradiation and stem cell technology may improve transplantation efficacy and targeting of MSCs, and enhance the efficacy of a sustained myocardial cell delivery. Ghanem *et al.*[69] demonstrated that focused ultrasound with stimulated microbubbles improved transplantation efficacy and allowed targeted engraftment of MSCs. Compared to nontargeted areas, significantly more MSCs adhered to the endothelium of targeted tissues was observed, and they did not observe any apoptosis phenomenon and/or myocardial necrosis. Otani *et al.*[70] proved the feasibility of combination of ultrasound and microbubbles for delivery of siRNA into MSC, and the results indicated that ultrasound and microbubble could serve as a nonviral delivery method of siRNA into MSC, which would be a useful appoach for regenerative medicine in the future.

**Figure 12:**
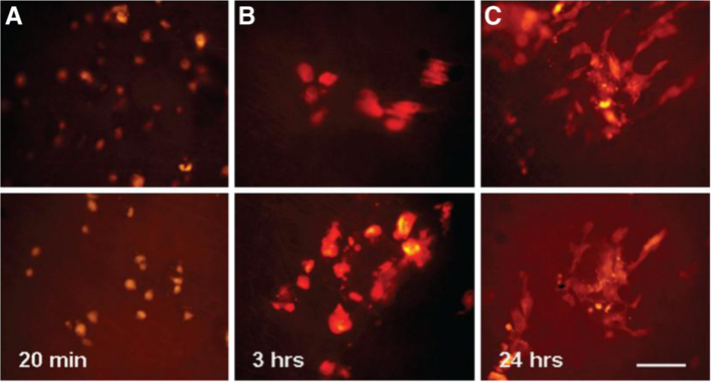
**The MSCs adherent to the aortic endoluminal surface after ultrasound mediated delivery survive and undergo morphological changes (Scale bar = 100 mm), between 20min (two examples in A) 3h (B), and 24 h (C) post delivery [**[71]**].**

Moreover, MSCs conld be surface-coated electrostatically with gas-filled lipid microbubbles (MB-MSCs) and seeded to targeted areas or a specific vascular segment, which is an emerging therapeutic option. Xu *et al.*[56] launched experimental research to figure out whether combining lipid-coated microbubbles with diagnostic US could enable the site directed delivery of MSCs into the myocardium even myocardial infarcted rabbits. After the intravenous injection of lipid-coated microbubble accompany with BM-MSCs into the rabbits, the anterior chest was treated with diagnostic ultrasound for 10 min to induce infusion of BM-MSCs which was labeled with DAPI in the nucleus. The results showed that DAPI positive cells in myocardial infarction area were much more than that of the MSCs infusion group (Figure 11). Toma *et al.*[71] proposed a novel method by using ultrasound-generated acoustic radiation force combined with MB-MSCs to delivery of therapeutic cells to a specific endovascular treatment site. This approach may be used for endoluminal cellular paving, providing a powerful tool for cell-based gene delivery of injured arterial segments (Figure 12).

## Conclusions

Increasing evidences prove that UTMD is a promising strategy to improve delivery efficiency, thus emerges as a method with great promise for target-specific gene delivery. A variety of experiments have demonstrated that the combination of UTMD and viral and non-viral vectors in gene delivery could not only enhance the efficiency of the viral vector, but also avoid its immunogenicity. Thus it may become a feasible, novel candidate for gene therapy, providing support to gene therapy trial for patients with cardiovascular diseases.

UTMD is a promising technique for gene delivery, but most of its studies are in the preclinical stage. UTMD still remains limited by its safety and efficiency. Future work needs to be done before its clinical application, including optimization of microbubble preparation technology to efficiently carry gene payloads while maintaining acoustic activity, prolonging circulation time to prevent clearance by the mononuclear cell, improving targeting techniques to enhance tissue binding force in areas of high shear stress, and illustration of optimal ultrasound parameters for each microbubble and its intended application. What is more important, as UTMD mediated gene therapy involve the multiple interacting modalities, there needs to be close collaboration between chemists, ultrasound engineers, and biologists to move this strategy to fruition.

## Competing interests

The authors declare no competing interests.

## Authors’ contributions

ZYC and SPG are responsible for designing the framework of this article. YL and FY participated in collecting material and drafting the manuscript. LJ participated in picture processing. All authors read and approved the final manuscript.
